# Protocol for validating liquid-liquid phase separation as a driver of membraneless organelle assembly *in vitro* and in human cells

**DOI:** 10.1016/j.xpro.2024.103410

**Published:** 2024-10-23

**Authors:** Marius Hedtfeld, Andrea Musacchio

**Affiliations:** 1 Department of Mechanistic Cell Biology, Max Planck Institute of Molecular Physiology, Otto-Hahn-Straße 11, 44227 Dortmund, Germany; 2 Centre for Medical Biotechnology, Faculty of Biology, University Duisburg-Essen, Essen, Germany

**Keywords:** Cell Biology, Protein Biochemistry, Protein expression and purification

## Abstract

Liquid-liquid phase separation (LLPS) of scaffold proteins has often been proposed to drive the biogenesis of membraneless cellular compartments. Here, we present a protocol to link *in vitro* LLPS propensity to localization *in vivo*. We describe steps for examining LLPS *in vitro* in the presence of crowding agents or cytomimetic media. We complement our *in vitro* studies with recombinant proteins with experiments of protein electroporation into mitotic HeLa cells. In addition, we discuss steps to assess protein localization and delivery levels.

For complete details on the use and execution of this protocol, please refer to Hedtfeld et al.^1^

## Highlights

• Detailed protocol to purify CPC targeting modules devoid of contaminating DNA
• Guide to test LLPS propensity of purported LLPS scaffolds *in vitro*
• Detailed protocol to deliver recombinant proteins in living cells by electroporation
• Instructions to analyze localization and delivery levels of electroporated proteins

## Abstract (editor highlights)

Publisher’s note: Undertaking any experimental protocol requires adherence to local institutional guidelines for laboratory safety and ethics.

## Graphical abstract

**Figure:**
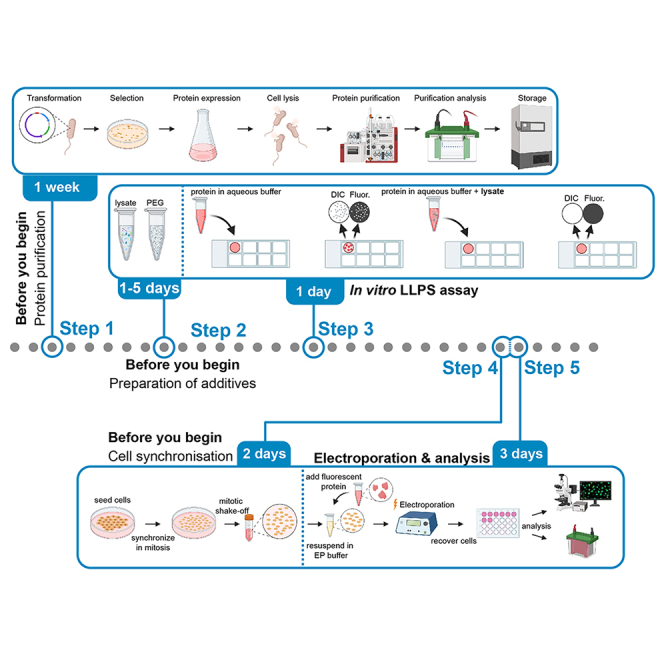

## Before you begin

In recent years, *in vitro* liquid-liquid phase separation (LLPS) assays have been widely used to identify potential scaffolds of LLPS and to implicate them as drivers in the formation of membraneless subcellular compartments. For LLPS of a single macromolecular species in a given solvent to occur, its concentration must exceed its saturation concentration, a condition in which scaffold-to-scaffold interactions outweigh those with solvent. LLPS scaffolds are often identified by homotypic or simple heterotypic *in vitro* phase separation assays, often in combination with crowding agents expected to mimic the cellular environment.

Here, we provide a step-by-step protocol to evaluate the predictive power of homotypic *in vitro* LLPS assays. We applied our validation pipeline to the Chromosomal Passenger Complex (CPC), a four-subunit complex comprising Survivin, Borealin, INCENP, and the catalytic protein kinase subunit Aurora B. During mitosis, the CPC promotes chromosome bi-orientation. CPC centromere recruitment, retention and function had been shown to depend on specific interactions with modified histones,^2^^,^^3^^,^^4^^,^^5^^,^^6^^,^^7^^,^^8^^,^^9^^,^^10^^,^^11^^,^^12^^,^^13^^,^^14^^,^^15^^,^^16^^,^^17^^,^^18^^,^^19^ but more recently work also implicated LLPS^20^^,^^21^ in this process. Using assays described here, we conclude that *in vitro* LLPS of the CPC is an unreliable predictor of its centromere localization. We show that LLPS of the CPC *in vitro* is enhanced by commonly used crowding agents, but that addition of more realistic cytomimetic media dissolves CPC droplets. Likewise, LLPS of several other proteins in aqueous buffers is effectively inhibited by cytomimetic media. Thus, our strategy can be adapted to evaluate the contribution of LLPS to the formation of membraneless compartments of a variety of cellular proteins.

### Expression and purification of CPC complexes

**Timing: 1 week**

**Timing: Day 1 (for steps 1a–g)**

**Timing: Day 2 (for step 1h)**

**Timing: Day 3 (for steps 1i and 1j)**

**Timing: Day 4 (for steps 2a–m)**

**Timing: Day 5 (for steps 2n–s)**

**Figure 1:**
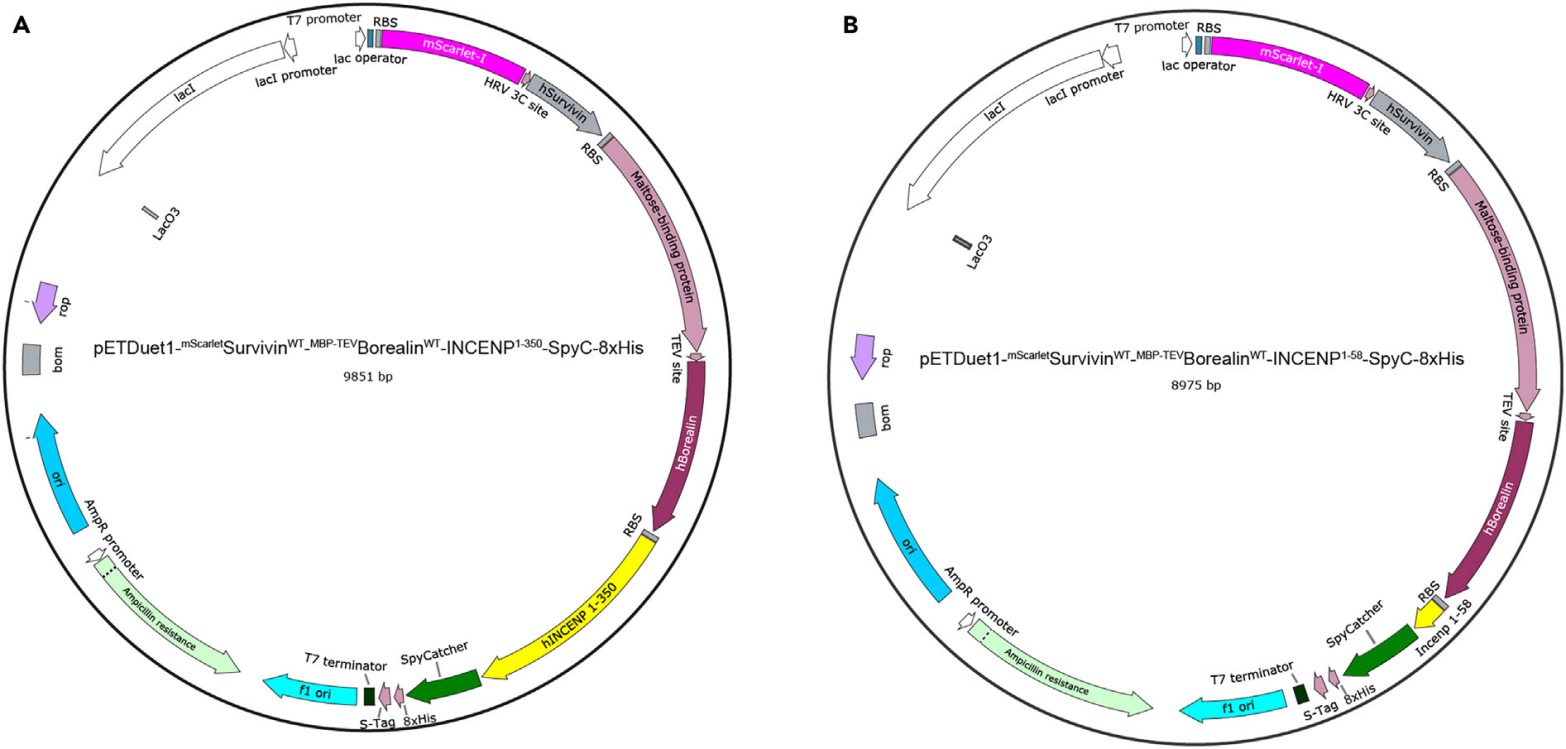
Plasmid maps of ^mScarlet^CPC-TARG^WT^ and ^mScarlet^ISB^WT^ (A and B) SnapGene-exported representation of pET-Duet vectors for expression of ^mScarlet^CPC-TARG^WT^ and ^mScarlet^ISB^WT^. Colored arrows mark the coding sequences of proteins as indicated by their labelling. RBS, ribosome binding site.

The following procedure details steps to express and purify CPC variants. Specifically, we describe the expression and purification of wild-type mScarlet-tagged CPC-TARG (for targeting CPC module) and ISB (for INCENP, Survivin, Borealin),^1^ which contain all the necessary determinants of localization, as well as mutants defective in centromere targeting. CPC-TARG and ISB differ in the length of INCENP. The ISB contains INCENP^1–58^ whereas CPC-TARG contains INCENP^1–350^. Both constructs have previously been shown to localize to centromeres. The pDuet expression vectors for wild-type ^mScarlet^CPC-TARG and ^mScarlet^ISB are shown in Figures 1A and 1B. Mutants of these complexes described in Hedtfeld et al. 2024 were derived from these constructs. Our protocol also aims to reduce the high initial level of DNA contamination after bacterial expression and lysis. We anticipate that our purification protocol will be effective for other His-tagged proteins with high initial levels of DNA contamination.

**Figure 2:**
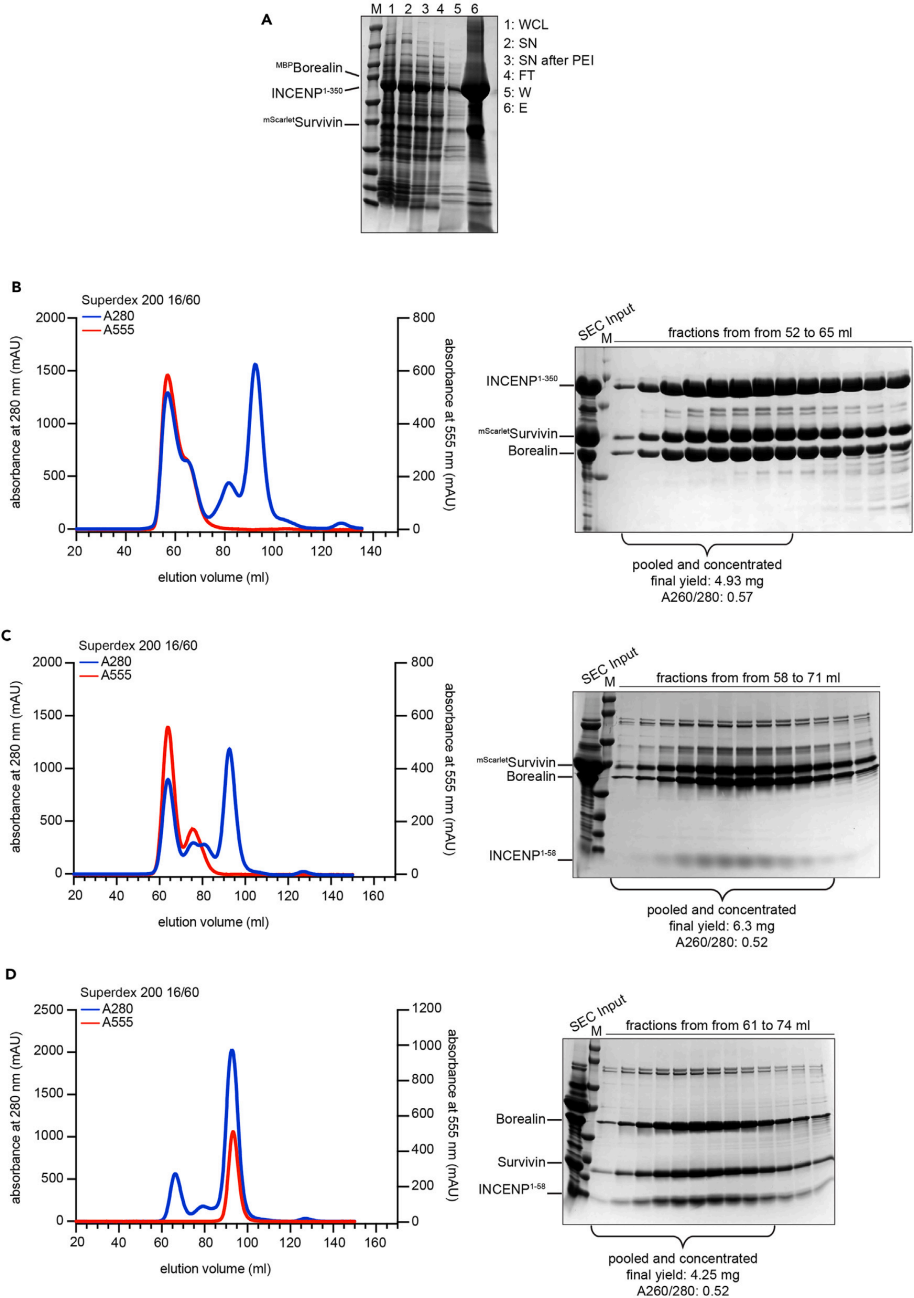
Representative SEC runs of ^mScarlet^CPC-TARG, ^mScarlet^ISB and ISB (A) SDS-PAGE of control samples taken during purification of ^mScarlet^CPC-TARG (steps 2d–k). WCL, whole cell lysate; SN, supernatant; SN after PEI, supernatant after PEI treatment; FT, flow-through; W, wash; E, elution. (B–D) Representative size-exclusion chromatography (SEC) runs and corresponding SDS-PAGE analysis of ^mScarlet^CPC-TARG^WT^ (B), ^mScarlet^ISB^WT^ (C), and ISB^WT^ (D). SEC input samples were taken immediately before injecting the protein (step 2p).

1. Expression of CPC constructs in BL21 CodonPlusRIL *E. coli* cells.
  a. Thaw a tube of BL21 CodonPlusRIL *E. coli* on ice.
  b. Add 50–100 ng of expression vector directly to the bacterial slurry.
  c. Incubate bacteria + plasmid on ice for 5 min.
  d. Heat shock bacterial cells at 42°C for 45 s.
  e. Place tubes back on ice and recover for 10 min.
  f. Add 600 μL of fresh LB medium without antibiotics and incubate at 37°C with 140 rpm shaking for 30 min.
  g. Plate 10 μL of bacteria slurry onto agar plates supplemented with 100 μg/mL ampicillin and 34 μg/mL chloramphenicol and incubate 16–20 h at 37°C.
  h. Pick a single colony to inoculate a 50 mL pre-culture of LB medium supplemented with 100 μg/mL ampicillin and 34 μg/mL chloramphenicol and incubate 16–20 h at 37°C and 140 rpm shaking.
  i. Inoculate 10 mL of the pre-culture into 1 L TB medium supplemented with 100 μg/mL ampicillin and 34 μg/mL chloramphenicol and grow bacteria at 37°C and 140 rpm shaking to an OD_600_ = 0.6. This will take approximately 3–4 h.
    ***Note:*** Remember to intermittently measure the OD to prevent bacteria from overgrowing.
  j. Add IPTG at a final concentration of 100 μM to induce protein expression and incubate the expression culture for 16–20 h at 18°C and 140 rpm shaking.
2. Harvesting of bacterial expression culture and protein purification (Figure 2).
  a. Harvest bacterial cells by centrifugation: 5000 × *g* for 20 min.
    **Pause point:** At this step the pellet can be flash-frozen in liquid nitrogen and stored at - 80°C before proceeding with the purification.
  b. Resuspend pellet in 5 pellet volumes of lysis buffer (see materials and equipment) and transfer to a beaker placed on ice.
  c. Add PMSF (c_final_ = 1 mM), TCEP (c_final_ = 1 mM), DNase I (c_final_ = 0.1 mg/mL) and MgCl_2_ (c_final_ = 5 mM).
  d. Lyse cells by sonication: 1 s on, 3 s off, amplitude of 60% (output power 240 W) for a total time of 90 s. Repeat the sonication once.
    i. Take sample for SDS-PAGE: 20 μL + 20 μL 5x SDS loading buffer (whole cell lysate = WCL).
      **CRITICAL:** Keep bacterial cell suspension on ice during the whole sonication process.
      **CRITICAL:** All following steps should be carried out at 4°C.
  e. Clarify lysate by ultracentrifugation: 100,000 × *g* for 30 min.
    **CRITICAL:** Use high-speed centrifuge tube, e.g., from Nalgene (see key resources table).
    i. Take sample for SDS-PAGE: 20μL + 20 μL 5x SDS loading buffer (supernatant = SN).
  f. Transfer the supernatant to a beaker and precipitate DNA by adding PEI (c_stock_ = 10%) droplet-wise to a final concentration of 0.25%. Incubate for 10 min at 4°C with mild stirring.
  g. Clarify lysate by ultracentrifugation: 100,000 × *g* for 30 min.
    i. Take sample for SDS-PAGE: 20 μL + 20 μL 5x SDS loading buffer (Supernatant after PEI = SN after PEI).
      **CRITICAL:** Use high-speed centrifuge tube, e.g., from Nalgene (see key resources table).
  h. Equilibrate 5 mL His-Trap column with 10 column volumes (CV; 50 mL) lysis buffer.
    **CRITICAL:** Always check in which buffer the affinity column is stored prior to use. If stored in 20% ethanol, the column must be washed with 10 CV filtered (0.2 μm) ddH_2_O before equilibration.
  i. Load supernatant onto a 5 mL His-Trap column.
    i. Collect the flow-through and take sample for SDS-PAGE: 20 μL + 20 μL 5x SDS loading buffer (Flow-through = FT).
  j. Wash the column with 10 CV of lysis buffer.
    i. Collect the flow-through and take sample for SDS-PAGE: 40 μL + 10 μL 5x SDS loading buffer (Wash = W).
  k. Elute bound protein in 10 CV (50 mL) of Ni-elution buffer.
    i. Take sample for SDS-PAGE: 40 μL + 10 μL 5x SDS loading buffer (Elution = E).
  l. Run an SDS-PAGE to analyze the control samples taken during the previous steps and the elution by Coomassie Brilliant Blue staining. Load the following volumes: 5 μL for WCL, SN and FT; 20 μL for W, E (Figure 2A).
  m. Cleave MBP-tag by adding 0.05 mg of TEV per 1 mg of protein and incubate at 4°C for 16–20 h with mild rotation.
    i. Take sample for SDS-PAGE: 40 μL + 10 μL 5x SDS loading buffer (Elution after TEV cleavage = E after TEV).
      ***Note:*** The amount of TEV to be added depends on the activity of the protease. We use the TEV protease at 0.05 mg per 1 mg of target protein. You may need to recalculate the amount according to the activity of the available TEV.
      ***Note:*** Similar to step 2m, the N-terminal fluorescent tag of Survivin can be removed by 3C PreScission protease cleavage (Figure 2C).
  n. Equilibrate a 100 kDa cut-off Amicon concentrator with Ni-elution buffer.
    i. Add 4 mL of buffer and spin for 10 min at 4000 × *g*.
      **CRITICAL:** The 100 kDa cut-off is specifically chosen because of the molecular weight of the ^mScarlet^CPC-TARG (∼120 kDa) and needs to be changed depending on the molecular weight of the protein of interest.
  o. Concentrate TEV-digested protein solution to a final volume of 3 mL.
    i. Fill equilibrated Amicon concentrator with TEV-digested protein solution and spin for 10 min at 4000 × *g*.
    ii. Resuspend protein solution to prevent protein aggregation at the bottom of the concentrator.
    iii. Repeat steps i-ii until the target volume is reached.
      ***Note:*** The desired target volume depends on the size-exclusion column and the loop used for injection. To avoid loss of protein during the purification, as a rule of thumb, we recommend to inject a final volume not greater than 60% of the loop volume.
  p. Spin concentrated sample in a table top centrifuge at 20,000 × *g* for at least 10 min.
    i. Take Input sample for SDS-PAGE: 20 μL + 20 μL 5x SDS loading buffer (= SEC Input).
  q. Subject concentrated protein solution to size-exclusion chromatography (SEC; S200 16/60 column) by injecting into a 5 mL loop.
    ***Note:*** Size-exclusion chromatography separates proteins and protein complexes depending on their size and shape. Thus, the type of column needs to be carefully chosen based on the expected molecular weight and shape.
  r. Analyze fractions of SEC eluate by SDS-PAGE and pool fractions containing protein of interest (Figures 2B–2D).
    i. Take fraction sample for SDS-PAGE: 40 μL + 10 μL 5x SDS loading buffer.
    ii. Load 20 μL of each fraction and 5 μL of SEC Input.
  s. Concentrate the protein to the desired concentration, flash-freeze in liquid nitrogen and store at − 80°C until further use.

### Preparation of whole cell lysates, additives, and crowding agents

**Timing:Variable**

**Timing: 2 days (for step 3)**

**Timing: ∼1 week (for step 4)**

**Timing: Day 1 (for steps 4a–d)**

**Timing: Day 2 (for steps 4e and 4f)**

**Timing: Day 3 (for steps 4g–k)**

**Timing: 1 day (for step 5)**

Examination of LLPS propensities in the context of macromolecular crowding is important to assess purported LLPS scaffolds. The following section outlines detailed procedures for preparing cytomimetic media from bacteria and mammalian cells depleted of endogenous CPC, additives and polymeric crowding agents.

3. Preparation of whole cell bacterial lysate.
  a. Thaw a tube of BL21 CodonPlusRIL *E.coli* on ice.
  b. Use 2 μL to inoculate a 100 mL pre-culture and grow bacteria for 14 h at 37°C and 140 rpm shaking.
  c. Inoculate 10 mL of the pre-culture into 1 L of TB medium and grow bacteria for 14 h at 37°C and 140 rpm shaking.
  d. Harvest bacteria by centrifugation at 5000 × *g* for 20 min.
    **Pause point:** At this step the pellet can be flash-frozen in liquid nitrogen and store at - 80°C.
  e. Resuspend pellet in lysis buffer (see materials and equipment).
    ***Note:*** To yield a highly concentrated whole cell lysate resuspend the pellet in as little lysis buffer as possible.
  f. Lyse cells by sonication: 1 s on, 3 s off, amplitude of 60% (output power 240 W) for a total time of 90 s. Repeat the sonication once.
  g. Clarify lysate by ultracentrifugation at 100,000 × *g* for 30 min.
    **CRITICAL:** Use high-speed centrifuge tube, e.g., from Nalgene (see key resources table).
  h. Measure total protein concentration of the resulting clarified, whole cell bacterial lysate using the 1 Abs = 1 mg/mL option of a Nanodrop2000 device.
    ***Alternative:*** The total protein concentration can be measured using Bradford or BCA protein assays.
    ***Optional:*** The lysate may be concentrated using a small cut-off Amicon concentrator, e.g., 3 kDa cut-off.
  i. Prepare aliquots of 10–20 μL, flash-freeze in liquid nitrogen and store at - 80°C until further use.
    **CRITICAL:** Use the whole cell lysate only once and avoid repeated freeze-thaw cycles of the aliquots.
4. Preparation of whole cell mammalian lysate depleted of endogenous CPC.
  a. If needed: Follow steps 6a-e to thaw fresh HeLa cells from frozen stocks.
    ***Note:*** We suggest to thaw cells for electroporation approximately 1 week prior to the start of the experiment to allow cells to recover from the thawing process.
  b. Perform reverse RNAi transfection of siRNA oligos directed against each CPC subunit (c_final_ = 10 nM) using RNAiMax transfection reagent following the manufacturers’ instructions (https://www.thermofisher.com/de/en/home/references/protocols/cell-culture/transfection-protocol/rnaimax-reverse-transfections-lipofectamine.html).
    i. Aspirate old complete DMEM with a vacuum pump.
    ii. Wash cells with 3 mL of PBS pre-warmed to 37°C.
    iii. Add 1 mL of 0.05% Trypsin/EDTA, gently distribute the Trypsin and place in a 37°C incubator.
    iv. For each reaction, prepare a siRNA oligo mastermix of 160 nM in a final volume of 500 μL OptiMEM in one well of a 12-er well plate.
    v. For each reaction, prepare a RNAiMax Lipofectamine mastermix by adding 15 μL RNAiMax to a total volume of 500 μL OptiMEM in one well of a 12er well plate.
    vi. Mix solution from steps iv and v 1:1 and incubate 5 min.
    vii. During the incubation time, dilute cells in complete DMEM (max. volume = 7 mL) so that they reach a confluency of 40%–50% after plating.
    viii. Fill volume of complete DMEM to 7 mL if required.
    ix. Add 1 mL of siRNA/ RNAiMax mix from step vi to each dish.
      ***Note:*** The volumes of OptiMEM, RNAiMax and complete DMEM used for cell resuspension refer to a 10-cm tissue culture dish and may need to be adjusted and optimized for other formats.
  c. Place cells in an appropriate cell culture incubator (37°C, 5% CO_2_ and humidified environment) for 6–8 h and check intermittently with the microscope that all cells have attached.
  d. To synchronize cells at the G1-/S-phase transition, add thymidine (c_final_ = 2 mM) directly to each cell culture dish and treat cells for 18 h.
  e. Washout thymidine by carefully washing the cells 3x with PBS pre-warmed to 37°C. This allows cells to resume cycling.
    i. Aspirate old complete DMEM with a vacuum pump.
    ii. Add 25 mL of PBS.
    iii. Aspirate freshly added PBS with a vacuum pump.
    iv. Repeat steps i-iii two more times.
  f. Place cells in an appropriate cell culture incubator (37°C, 5% CO_2_ and humidified environment) for 6 h, then add RO3306 (c_final_ = 9 μM) and treat cells for 18–20 h.
    ***Note:*** RO3306 is a CDK1 inhibitor. This step arrests cells in G2-phase and subsequent RO3306 washout allows cells to rapidly and synchronously enter mitosis.
  g. Harvest cells by trypsinization.
    i. Aspirate old complete DMEM with a vacuum pump.
    ii. Wash cells with 3 mL of PBS pre-warmed to 37°C.
    iii. Add 1 mL of 0.05% Trypsin/EDTA, gently distribute the Trypsin and place in a 37°C incubator for 5 min.
    iv. Check under the microscope if all cells have detached from the surface.
    v. Resuspend detached cells in 8 mL complete DMEM and collect all cells by gently pipetting up and down several times.
    vi. Transfer to a 50 mL Falcon tube and fill up to 50 mL with PBS pre-warmed to 37°C.
    vii. Centrifuge at 500 × *g* for 5 min and discard the supernatant.
    viii. Resuspend the pellet in 2 mL PBS pre-warmed to 37°C, transfer cell suspension into a 2 mL Eppendorf tube and repeat step vii.
  h. Resuspend pellet in one pellet volume lysis buffer and lyse cells.
    i. Flash-freeze cell suspension in liquid nitrogen for at least 30 s.
    ii. Remove Eppendorf tube containing the frozen cell suspension and thaw it on ice. Depending on the pellet size this takes at least 5–10 min.
    iii. Repeat steps i-ii twice.
    iv. Sonicate using the BioRapture sonicator: 20 cycles of 10 s on/ 30 s off at high power (the amplitude of this setting is indicated at 4 mA and approximately 300 W) and 4°C.
  i. Clarify lysate by ultracentrifugation at 75,000 × *g* for 90 min.
    **CRITICAL:** Use high-speed centrifuge tube, e.g., from Beckman Coulter (see key resources table).
  j. Transfer supernatant to a new tube and measure protein concentration using the 1 Abs = 1 mg/mL option of a NanoDrop2000.
  k. Flash-freeze in liquid nitrogen and store at - 80°C until further use.
5. Preparation of additives and crowding agents.
  ***Note:*** FBS was purchased from PAN-Biotech, filtered and added directly to the *in vitro* LLPS reaction without additional preparation.
  a. Dissolve PEG3350 and Ficoll400 in ddH_2_O to a concentration of 50% (w/v).
    i. Weigh the appropriate amount (see materials and equipment) of the polymers and gently stir until they are completely dissolved.
  b. Dissolve lysozyme and Bovine Serum Albumin (BSA) in NO salt size -exclusion buffer to make a stock solution at a concentration of 150 mg/mL.
    i. Weigh the appropriate amount (see materials and equipment) and gently stir until they are completely dissolved.

### Cell preparation for electroporation

**Timing: 7–9 days**

The following section describes the preparation of mitotic HeLa cells for electroporation. We refer only to the preparation of otherwise untreated cells, but this approach can be coupled with RNAi-mediated depletion or drug treatments.

6. Thawing of frozen HeLa cell stocks.
  a. Retrieve a frozen vial of HeLa cells from the long-term storage space.
  b. Rapidly thaw frozen cells in a 37°C water bath.
  c. Transfer cell slurry to a 15 mL Falcon tube pre-filled with complete DMEM pre-warmed to 37°C.
  d. Centrifuge the cell suspension for 5 min at 500 × *g* and aspirate the supernatant.
  e. Resuspend cell pellet in 8 mL of complete DMEM and transfer to a 10-cm cell culture dish.

***Note:*** We suggest to thaw cells for electroporation approximately 1 week prior to the start of the experiment to allow cells to recover from the thawing process.

**CRITICAL:** Always perform the electroporation on a relatively fresh batch of HeLa cells (maximally cultured for ∼1 month). Do not keep your cells in culture for an extended period of time as this can negatively affect electroporation efficiency.

**Figure 3:**
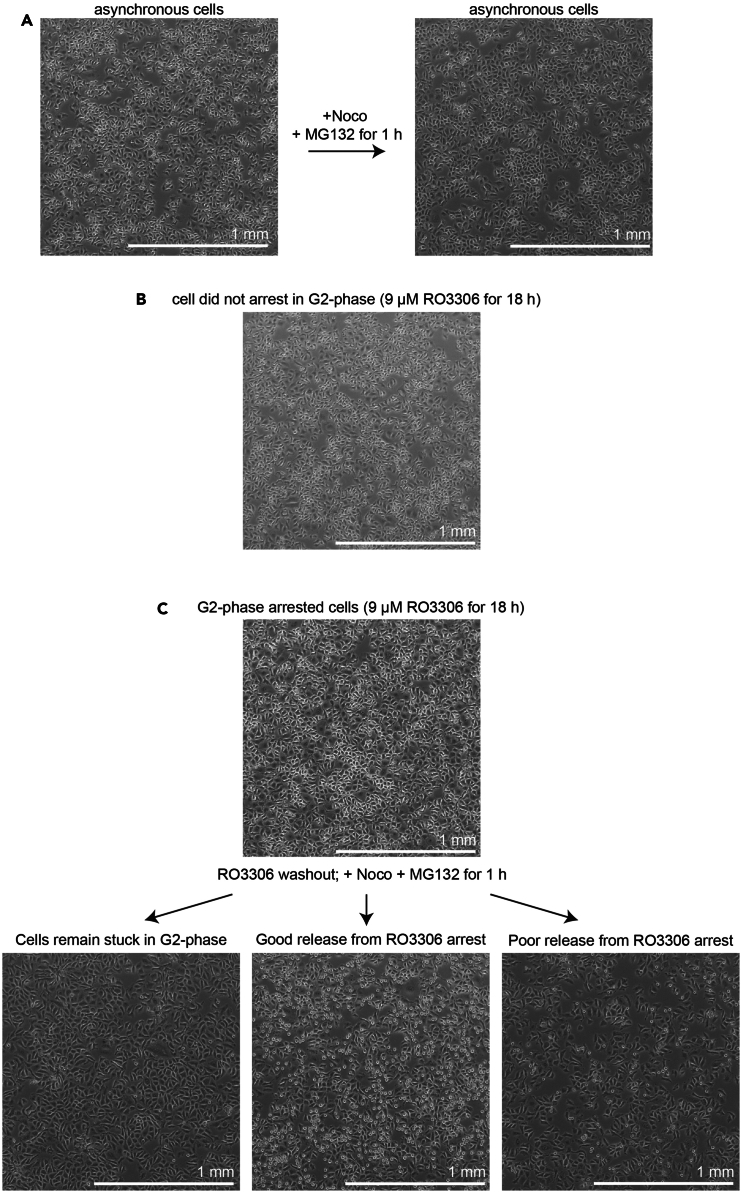
Comparison of RO3306 arrest and release efficiency (A) Representative images of asynchronous growing HeLa cells treated with Nocodazole (3.3 μM) and MG132 (10 μM) for 1 hour. (B) Representative images of HeLa cells that were treated with the CDK1 inhibitor RO3306 (9 μM) for 18 hours. (C) Representative images of HeLa cells that were released from an 18-hour G2-phase arrest (RO3306, 9 μM) into Nocodazole (3.3 μM) and MG132 (10 μM) for 1 hour. Scale bar in all panels: 1 mm.
  d. Wash out RO3306 with complete DMEM pre-warmed to 37°C.
    i. Aspirate old complete DMEM with a vacuum pump.
    ii. Add 25 mL of fresh complete DMEM.
    iii. Aspirate freshly added complete DMEM with a vacuum pump.
    iv. Repeat steps i-iii four times.
      **CRITICAL:** Ensure sufficient washing to completely remove the RO3306 and allow an efficient release of cells from the G2-arrest into mitosis. (Figure 3C).
  e. Add complete DMEM supplemented with Nocodazole (c_final_ = 3.3 μM) and MG132 (c_final_ = 10 μM) and place the dishes back in the incubator.
    ***Note:*** For preparing cells arrested in prometaphase, Nocodazole will be sufficient and addition of MG132 optional. However, if treatments that interfere with the Spindle Assembly Checkpoint are included, MG132 must be added to prevent mitotic exit.
    ***Alternatives:*** Other drugs, e.g., STLC (c_final_ = 5 μM), can be used to arrest and enrich cells in prometaphase.
  f. After ∼1 h check the cells under the microscope and assess the percentage of mitotic cells. Problem 1.
    ***Note:*** In our hands 1 h is sufficient to enrich the majority of cells in mitosis. However, the efficiency strongly depends on the RO3306 washout (Figure 3C). If the RO3306 release is less efficient it can be extended to 2 h.
  g. Proceed with electroporation (see electroporation of recombinant proteins into living cells).

7. Cell seeding and mitotic arrest.
  a. Seed HeLa cells 1 day prior to electroporation in a 10-cm cell culture dish at a confluency of ∼70%.
    i. Aspirate old complete DMEM with a vacuum pump.
    ii. Wash cells with 3 mL of PBS pre-warmed to 37°C.
    iii. Add 1 mL of 0.05% Trypsin/EDTA, gently distribute the Trypsin and place in an incubator at 37°C and 5% CO_2_ in a humid environment for 5 min.
    iv. Check under the microscope that all cells have detached from the plate.
    v. Resuspend detached cells in complete DMEM and collect all cells by gently pipetting up and down several times.
    vi. Add the appropriate volume of the cell suspension to a new 10-cm cell culture dish.
  b. Recover freshly seeded cells until the next day.
  c. Add RO3306 (c_final_ = 9 μM) directly to each cell culture dish and place the dish back in the incubator for 16–20 h.
    ***Note:*** Cells that are well arrested should appear enlarged and relatively flat with, ideally, no mitotic cells (Figure 3C, top). If the RO3306 arrest did not work, cells grow indistinguishably from asynchronously growing cells (compare Figure 3A left, and 3B).

## Key resources table

**Table:** 

REAGENT or RESOURCE	SOURCE	IDENTIFIER
**Antibodies**		

Anti-Aurora B, rabbit polyclonal (1/1,000 for immunofluorescence and western blotting)	Abcam	RRID: AB_302923
Anti-Aurora B, mouse monoclonal (1/100 for immunofluorescence)	Abcam	RRID: AB_449204
anti-CENP-C, guinea pig polyclonal (1/1,000 for immunofluorescence)	MBL (via Biozol)	RRID: AB_10693556
Anti-INCENP, mouse monoclonal (1/5,000 for immunofluorescence)	Invitrogen	RRID: AB_2804171
Anti-Survivin, rabbit monoclonal (1/1,000 for immunofluorescence and western blotting)	Cell Signaling Technology	RRID: AB_2063948
Anti-vinculin, mouse monoclonal (1/10,000 for western blotting)	Sigma	RRID: AB_477629
Anti-rabbit IgG Alexa 488 secondary (1/200 for immunofluorescence)	Invitrogen	RRID: AB_2535792
Anti-guinea pig IgG Alexa 647 secondary (1/200 for immunofluorescence)	Invitrogen	RRID: AB_141882
Anti-rabbit IgG HRP (1/10,000 for western blotting)	Cytiva (GE)	RRID: AB_772206
Anti-mouse IgG HRP (1/10,000 for western blotting)	Amersham	RRID: AB_772209

**Bacterial and virus strains**		

*E. coli*: BL21CodonPlus(DE3)-RIL strain	Agilent Technologies	Cat#230240
One Shot OmniMax 2T1R	Invitrogen	#C854003

**Chemicals, peptides, and recombinant proteins**		

RO3306	Millipore	Cat#217699
MG132	Calbiochem	CAS 133407-82-6
Thymidine	Sigma	Cat#T9250
Microcystin	Enzo	Cat# ALX-350-012-C100
Nocodazole	Sigma	Cat#M1404
16% PFA	Alfa Aesar	Cat#11400580
TCEP	Sigma	Cat#75259
PMSF	Sigma	Cat#78830-1G
Polyethyleneimine (PEI)	Sigma	P3143-100mL
Ponceau S	Thermo Fisher Scientific	Cat#A40000279
IPTG	Sigma	Cat#16758
Lipofectamine RNAiMax	Invitrogen/Thermo Fisher Scientific	Cat#13778-150
FBS	Sigma	Cat#F7524
Normal goat serum	Histoprime via Biozol	Cat#LIN-EN9010
DMEM	PAN-Biotech	Cat#P04-03600
Opti-MEM	Life Technologies	Cat#31985-047
PEG3550	Jena Bioscience GmbH	Cat#800082
Ficoll 400	VWR	Cat#E965-50G
0.05% Trypsin/EDTA	PAN-Biotech	Cat#P10-023100
Lysozyme	Fisher Scientific GmbH	Cat#11374029
PBS	Produced in-house	N/A
BSA	VWR	Cat#422351S
L-glutamine	PAN-Biotech	Cat#P04-43100
DTE	Carl Roth	Cat#8814.1
DTT	Carl Roth	Cat#6908.2
Bromophenol blue	Serva	Cat#15375.2
ECL Prime western blotting detection reagent	Cytiva	Cat#RPN2232
NP-40	Thermo Fisher Scientific	Cat#85124
Milk powder	Carl Roth	Cat#T145.1
Mowiol	Calbiochem	Cat#475904
DAPI	Sigma	Cat#D9542
Triton X-100	Sigma	Cat#T8787
Tween 20	Thermo Fisher Scientific	Cat#85113
Poly-L-lysine	Sigma	P4832-50mL
PIPES	Sigma	Cat#P6757
HEPES	Sigma	Cat#H3375
Tris	Carl Roth	Cat#4855.1
MgCl_2_	Sigma	Cat#M8266
MgSO_4_	Sigma	Cat#800021
NaCl	Sigma	Cat#S9888
EGTA	VWR	Cat#0732-100G
Glycerol	Sigma	Cat#G5516
Imidazole	Sigma	Cat#I5513
TCEP	Sigma	Cat#75259
Protease inhibitor cocktail	Serva	Cat#39107
Coomassie Brilliant Blue R-250 dye	Thermo Fisher Scientific	Cat#20278
PMSF	Serva	Cat#32395.2
DNase I	Thermo Fisher Scientific	Cat#89836
^mScarlet^CPC-TARG^WT^	Hedtfeld et al.^1^	N/A
^mScarlet^CPC-TARG^KH/AA^	Hedtfeld et al.^1^	N/A
^mScarlet^CPC-TARG^DD/AA^	Hedtfeld et al.^1^	N/A
^mScarlet^CPC-TARG^ΔC^	Hedtfeld et al.^1^	N/A
^mScarlet^CPC-TARG^KH/AA-ΔC^	Hedtfeld et al.^1^	N/A
^mScarlet^CPC-TARG^DD/AA-ΔC^	Hedtfeld et al.^1^	N/A
^mScarlet^CPC-TARG^Δ139–160^	Hedtfeld et al.^1^	N/A
^mScarlet^ISB^WT^	Hedtfeld et al.^1^	N/A
^mScarlet^ISB^DD/AA^	Hedtfeld et al.^1^	N/A
^mScarlet^ISB^Δ139–160^	Hedtfeld et al.^1^	N/A
^mCherry^MPS1	Raisch et al.^22^	N/A
^EGFP^CENP-E^2070-C^	Ciossani et al.^23^	N/A
CENP-OPQUR complex	Pesenti et al.^24^	N/A
CENP-CHIKM complex	Weir et al.^25^	N/A
TEV Protease	Produced in-house	N/A
DNase I	Thermo Fisher Scientific	Cat#89836

**Critical commercial assays**		

NEON transfection kit (including buffers)	Thermo Fisher Scientific	Cat#10090314; https://www.fishersci.de/shop/products/invitrogen-neon-transfection-system-starter-pack-serum-compatible-300w/10090314

**Deposited data**		

Source data associated with primary research was deposited to Mendeley	https://doi.org/10.17632/srwv7hcwd5.1	N/A

**Experimental models: Cell lines**		

HeLa cells	IEO Milan	N/A

**Oligonucleotides**		

siBorealin 5′-AGGUAGAGCUGUCUGUUCAdTdT-3′	Klein et al.^26^	N/A
siSurvivin 5′- GAGGCTGGCTTCATCCACTdTdT-3′	Lens et al.^27^	N/A
siAurora B 5′- AACGCGGCACUUCACAAUUGA-3′	Ditchfield et al.^28^	N/A
siINCENP 5′- GGCUUGGCCAGGUGUAUAUdTdT-3′	van der Horst et al.^29^	N/A

**Recombinant DNA**		

pDuet-1	Novagen	Cat#71146
pETDuet1-^mScarlet^Survivin^WT^-^MBP-TEV^Borealin^WT^-INCENP^1–350^-SpyC-8xHis	Hedtfeld et al.^1^	N/A
pETDuet1-^mScarlet^Survivin^KH/AA^-^MBP-TEV^Borealin^WT^-INCENP^1–350^-SpyC-8xHis	Hedtfeld et al.^1^	N/A
pETDuet1-^mScarlet^Survivin^DD/AA^-^MBP-TEV^Borealin^WT^-INCENP^1–350^-SpyC-8xHis	Hedtfeld et al.^1^	N/A
pETDuet1-^mScarlet^Survivin^WT^-^MBP-TEV^Borealin^ΔC^-INCENP^1–350^-SpyC-8xHis	Hedtfeld et al.^1^	N/A
pETDuet1-^mScarlet^Survivin^KH/AA^-^MBP-TEV^Borealin^ΔC^-INCENP^1–350^-SpyC-8xHis	Hedtfeld et al.^1^	N/A
pETDuet1-^mScarlet^Survivin^DD/AA^-^MBP-TEV^Borealin^ΔC^-INCENP^1–350^-SpyC-8xHis	Hedtfeld et al.^1^	N/A
pETDuet1-^mScarlet^Survivin^WT^-^MBP-TEV^Borealin^Δ139–160^-INCENP^1–350^-SpyC-8xHis	Hedtfeld et al.^1^	N/A
pETDuet1-^mScarlet^Survivin^WT^-^MBP-TEV^Borealin^WT^-INCENP^1–58^-SpyC-8xHis	Hedtfeld et al.^1^	N/A
pETDuet1-^mScarlet^Survivin^DD/AA^-^MBP-TEV^Borealin^WT^-INCENP^1–58^-SpyC-8xHis	Hedtfeld et al.^1^	N/A
pETDuet1-^mScarlet^Survivin^WT^-^MBP-TEV^Borealin^Δ139–160^-INCENP^1–58^-SpyC-8xHis	Hedtfeld et al.^1^	N/A

**Software and algorithms**		

GraphPad Prism 9.0	GraphPad software, Inc.	https://www.graphpad.com
ImageJ2/Fiji 2.9.0/1.53t	NIH	https://imagej.net/
Slidebook	3i	N/A
Excel 16.78.3	Microsoft Corp.	https://www.microsoft.com/en-us/microsoft-365/excel
Image Lab 6.0.1	Bio-Rad	https://www.fishersci.de/shop/products/invitrogen-neon-transfection-system-starter-pack-serum-compatible-300w/10090314

**Other**		

Spinning disk confocal microscope	3i	N/A
8-well μ-chamber slide	ibidi GmbH	Cat#80807
ChemiDoc MP Imaging System	Bio-Rad	Cat#12003153
ÄKTA Purifier chromatography systems	GE Healthcare	N/A
Amicon concentrators (3 kDa)	Merck Millipore	Cat#UFC9003
Amicon concentrators (100 kDa)	Merck Millipore	Cat#UFC9100
BioRapture sonicator	Diagenode	Cat#B01020001
Centrifuge Allegra X-14	Beckman Coulter	Cat#A99464
Centrifuge tubes (high-speed)	Nalgene/Merck	Cat#T2918-10EA
Centrifuge tubes (high-speed)	Beckman Coulter	Cat#344062
Coverslips	Paul Marienfeld GmbH & Co. KG	Cat#0117520
Electrotransfer system for western blotting	Bio-Rad	Cat#1703930
His-Trap column (5 mL)	Cytiva	GE17-5248-01
Polyvinylidene fluoride (PVDF) membrane	GE Healthcare	Cat#88520
SDS-PAGE electrophoresis system	Bio-Rad	Cat#1658004
Superdex 200 16/60	GE Healthcare	Cat#28989335
Sonifier cell disruptor	Branson Ultrasonics	Cat#15549624
Table top centrifuge	Eppendorf	Cat#5420000318
TC20 automated cell counter	Bio-Rad	Cat#14501102
Ultracentrifuge Optima MAX-XP	Beckman Coulter	Cat#393315
Ultracentrifuge ProteomeLab XLA	Beckman Coulter	Cat#969342

## Materials and equipment

Reagents for protein expression and purification.

**Table:** 10% Polyethyleneimine (PEI) solution

Reagent	Final concentration	Amount
PEI (50%)	10%	2 mL
TRIS (1 M)	50 mM	0.5 mL
NaCl (5 M)	200 mM	0.4 mL
ddH_2_O	N/A	up to 10 mL
**Total**	**N/A**	**10 mL**

Store at 20°C–25°C wrapped in aluminum foil.

**CRITICAL:** PEI is toxic. All PEI contaminated waste must be disposed properly in the toxic waste. Always wear gloves, safety goggles and a lab coat when handling PEI.

**Table:** 0.5 M TCEP

Reagent	Final concentration	Amount
TCEP	0.5 M	35.9 g
ddH_2_O	N/A	up to 250 mL
**Total**	**N/A**	**250 mL**

Store at −20°C for up to 1 month.

***Note:*** Adjust pH with NaOH pellets to 7.

**Table:** 1 M Tris-HCl pH 6.8

Reagent	Final concentration	Amount
Tris	1 M	30.25 g
ddH_2_O	N/A	up to 500 mL
**Total**	**N/A**	**500 mL**

Filtered 1 M Tris-HCl can be stored at 20°C–25°C.

***Note:*** Adjust pH with 25% HCl to 6.8.

**Table:** 5x SDS-PAGE sample buffer

Reagent	Final concentration	Amount
Tris-HCl pH 6.8 (1 M)	250 mM	25 mL
SDS	10%	4 g
Glycerol (100%)	50%	50 mL
DTE	10 mM	3,86 g
Bromophenol Blue	0.05%	0.05 g
ddH_2_O	N/A	up to 100 mL
**Total**	**N/A**	**100 mL**

Can be stored at −20°C indefinitely.

**Table:** 1 M HEPES pH 7.5

Reagent	Final concentration	Amount
HEPES	1 M	238.3 g
ddH_2_O	N/A	up to 1 L
**Total**	**N/A**	**1 L**

Filtered 1 M HEPES can be stored at 20°C–25°C or 4°C for several weeks.

**Table:** 5M NaCL

Reagent	Final concentration	Amount
NaCl	5 M	292.2 g
ddH_2_O	N/A	up to 1 L
**Total**	**N/A**	**1 L**

Filtered 5 M NaCl can be stored at 20°C–25°C or 4°C indefinitely.

**Table:** 50% Glycerol

Reagent	Final concentration	Amount
Glycerol (100%)	50%	500 mL
ddH_2_O	N/A	up to 1 L
**Total**	**N/A**	**1 L**

Filtered 50% glycerol can be stored at 20°C–25°C or 4°C indefinitely.

**Table:** 1 M IPTG

Reagent	Final concentration	Amount
IPTG	1 M	23.8 g
ddH_2_O	N/A	up to 100 mL
**Total**	**N/A**	**100 mL**

Store IPTG at −20°C.

**Table:** Lysis buffer

Reagent	Final concentration	Amount
HEPES pH 7.5 (1 M)	50 mM	12.5 mL
NaCl (5 M)	500 mM	25 mL
Glycerol (50%)	5%	25 mL
Imidazole (3 M)	20 mM	1.66 mL
TCEP (0.5 M)	1 mM	0.5 mL
ddH_2_O	N/A	up to 250 mL
**Total**	**N/A**	**250 mL**

Store at 4°C for up to 1 week.

**Table:** Ni-elution buffer

Reagent	Final concentration	Amount
HEPES pH 7.5 (1 M)	50 mM	12.5 mL
NaCl (5 M)	500 mM	25 mL
Glycerol (50%)	5%	25 mL
Imidazole (3 M)	250 mM	21 mL
TCEP (0.5 M)	1 mM	0.5 mL
ddH_2_O	N/A	up to 250 mL
**Total**	**N/A**	**250 mL**

Store at 4°C for up to 1 week.

**Table:** Size-exclusion (SEC) buffer

Reagent	Final concentration	Amount
HEPES pH 7.5 (1 M)	50 mM	12.5 mL
NaCl (5 M)	500 mM	25 mL
Glycerol (50%)	5%	25 mL
TCEP (0.5 M)	1 mM	0.5 mL
ddH_2_O	N/A	up to 250 mL
**Total**	**N/A**	**250 mL**

Filtered SEC buffer can store at 4°C for up to 1 week, although we suggest preparing it freshly every time.

Reagents for mammalian cell culture, electroporation, immunofluorescence and western blotting.

**Table:** Complete DMEM

Reagent	Final concentration	Amount
DMEM	N/A	500 mL
L-Glutamine	1%	5.5 mL
FBS	10%	50 mL
**Total**	**N/A**	**555 mL**

Store at 4°C for up to 1 month.

***Note:*** Antibiotics such as Penicillin-Streptomycin can be added to a final concentration of 1%.

**Table:** 10x PBS

Reagent	Final concentration	Amount
NaCl	1.37 M	80 g
KCl	27 mM	2 g
KH_2_PO_4_	18 mM	2.4 g
Na_2_HPO_4_ ∗ 2 H_2_O	100 mM	17.8 g
ddH_2_O	N/A	up to 1 L
**Total**	**N/A**	**1 L**

PBS can be stored at 20°C–25°C for up to 1 year.

**Table:** 10% NP-40

Reagent	Final concentration	Amount
NP-40 (100%)	10%	1 mL
ddH_2_O	N/A	up to 10 mL
**Total**	**N/A**	**10 mL**

10% NP-40 can be stored at −20°C indefinitely.

**Table:** 0.5 M EGTA pH8

Reagent	Final concentration	Amount
EGTA	0.5 M	95 g
ddH_2_O	N/A	up to 500 mL
**Total**	**N/A**	**500 mL**

Filtered 0.5 M EGTA can be stored at room temperature indefinitely.

**CRITICAL:** EGTA only dissolves properly once the pH has been raised sufficiently. Before filling up to 500 mL adjust to pH = 8 with NaOH.

**Table:** 1 M DTT

Reagent	Final concentration	Amount
DTT	1 M	1.5 g
ddH_2_O	N/A	up to 10 mL
**Total**	**N/A**	**10 mL**

At −20°C DTT can be stored up to 3 months.

**Table:** Western Blot lysis buffer (completed)

Reagent	Final concentration	Amount
HEPES pH 7.5 (1 M)	75 mM	150 μL
MgCl_2_ (1 M)	1.5 mM	3 μL
KCl (3 M)	150 mM	100 μL
Glycerol (50%)	10%	400 μL
EGTA pH 8 (1 M)	1.5 mM	3 mL
NP-40 (10%)	0.1%	20 μL
DTT (1 M)	1 mM	2 μL
Protein inhibitor mix (500x)	2x	8 μL
ddH_2_O	N/A	up to 2 mL
**Total**	**N/A**	**2 mL**

Completed western blot lysis buffer can be stored at −20°C and re-used up to 3 times.

**Table:** 4x PHEM

Reagent	Final concentration	Amount
PIPES	120 mM	145.12 g
HEPES	50 mM	47.6 g
MgSO_4_	8 mM	3.84 g
EGTA	10 mM	15.2 g
ddH_2_O	N/A	up to 2 L
**Total**	**N/A**	**2 L**

Filtered 4x PHEM stock can be stored at −20°C indefinitely.

**CRITICAL:** EGTA only dissolves properly once the pH has been raised sufficiently. Before filling up to 2 L adjust to pH = 7 with KOH pellets.

**Table:** Pre-extraction solution

Reagent	Final concentration	Amount
Triton-X-100 (100%)	0.5%	10 μL
1x PHEM	N/A	up to 2 mL
**Total**	**N/A**	**2 mL**

Pre-extraction solution should not be stored and always be prepared freshly.

**Table:** Fixation solution

Reagent	Final concentration	Amount
PFA (16%)	4%	2.5 mL
1x PHEM	N/A	up to 10 mL
**Total**	**N/A**	**10 mL**

Fixation solution can be stored at −20°C.

**Table:** 5% Boiled Goat Serum (BGS)

Reagent	Final concentration	Amount
Normal Goat Serum (100%)	5%	10 mL
1x PHEM	N/A	up to 200 mL
**Total**	**N/A**	**200 mL**

5% BGS can be stored at −20°C.

**CRITICAL:** To prepare the BGS, the Normal Goat Serum must be boiled for 10 min. Afterward, let the solution cool down until you can touch the flask with the hands. Next, spin the solution in an ultracentrifuge for 30 min at 50,000 × *g* and 4°C. Filter (0.2 μm) and aliquot the supernatant.

**Table:** 2.5% BGS-PHEM-T

Reagent	Final concentration	Amount
BGS (5%)	2.5%	1 mL
1x PHEM	N/A	1 mL
Triton-X (100%)	0.1%	2 μL
**Total**	**N/A**	**2 mL**

Store at −20°C.

**Table:** 10x TBS

Reagent	Final concentration	Amount
Tris	200 mM	24 g
NaCl	1500 mM	88
ddH_2_O	N/A	up to 1 L
**Total**	**N/A**	**1 L**

10x can be stored at 20°C–25°C for up to 1 year.

**Table:** TBS-T

Reagent	Final concentration	Amount
TBS (10x)	1x	100 mL
Tween-20 (100%)	0.1%	1 mL
ddH_2_O	N/A	up to 1 L
**Total**	**N/A**	**1 L**

Freshly prepared TBS-T can stored at 20°C–25°C for up to 1 week.

**Table:** 5% milk in TBS-T

Reagent	Final concentration	Amount
milk powder	5%	2.5 g
TBS-T	N/A	50 mL
**Total**	**N/A**	**50 mL**

Store at 20°C–25°C.

Reagents for *in vitro* LLPS assay.

**Table:** Lysis buffer to prepare bacterial whole cell lysate

Reagent	Final concentration	Amount
HEPES pH 7.5 (1 M)	50 mM	2.5 mL
NaCl (5 M)	25 mM	0.25 mL
Glycerol (50%)	5%	5 mL
TCEP (0.5 M)	1 mM	0.1 mL
ddH_2_O	N/A	up to 50 mL
**Total**	**N/A**	**50 mL**

Store at 4°C for up to 1 week.

**Table:** 50% PEG3350

Reagent	Final concentration	Amount
PEG3350	50%	10 g
ddH_2_O	N/A	up to 20 mL
**Total**	**N/A**	**20 mL**

Store at 4°C.

**Table:** 50% Ficoll400

Reagent	Final concentration	Amount
Ficoll 400	50%	10 g
ddH_2_O	N/A	up to 20 mL
**Total**	**N/A**	**20 mL**

Store at 4°C.

**Table:** NO salt size-exclusion buffer

Reagent	Final concentration	Amount
HEPES pH 7.5 (1M)	50 mM	12.5 mL
Glycerol (50%)	5%	25 mL
TCEP (0.5 M)	1 mM	0.5 mL
ddH_2_O	N/A	up to 250 mL
**Total**	**N/A**	**250 mL**

Store at 4°C for up to 1 week.

**Table:** 150 mg/mL lysozyme

Reagent	Final concentration	Amount
Lysozyme	150 mg/mL	150 mg
NO salt size-exclusion buffer	N/A	up to 1 mL
**Total**	**N/A**	**1 mL**

Filtered solution can be stored at 4°C for up to 1 week.

**Table:** 150 mg/mL BSA

Reagent	Final concentration	Amount
BSA	150 mg/mL	150 mg
NO salt size-exclusion buffer	N/A	up to 1 mL
**Total**	**N/A**	**1 mL**

Filtered solution can be stored at 4°C for up to 1 week.

## Step-by-step method details

### *In vitro* LLPS assay

**Timing: 1 day (depending on the number of proteins tested,timing increases)**

This assay is designed to examine the propensity of recombinant proteins to undergo LLPS in solely aqueous buffer compared to buffer supplemented with lysates, additives or crowding agents.

1. Characterization of protein LLPS propensities.
  ***Note:*** Before starting the experiment, you may want to prepare the imaging system. This includes setting up the image acquisition parameters as well as the focal plane.
  **CRITICAL:** Perform the *in vitro* LLPS assay in a temperature-controlled environment/ room. The temperature between experiments performed on different days should not fluctuate too much.
  a. Calculate the volumes required to achieve the desired final protein and salt concentrations in a final volume of 35 μL.
  b. Set up reaction in a 0.5 mL Eppendorf tube.
    i. Add the appropriate volumes of protein as well as the SEC buffer.
    ii. Add the appropriate volume of “NO salt buffer” and mix by pipetting up and down 15x.
  c. Immediately add 30 μL of the solution into a well of an 8-well μ-chamber slide, start the timer.
  d. Immediately acquire an image (t = 0 min) using the transmission light channel of your microscope. If the protein of interest is fluorescently labeled also acquire the corresponding wavelength.
  e. Assess droplet formation, i.e., are droplets formed or does the protein remain homogenously in solution.
  f. Incubate 5 min.
    ***Note:*** The incubation time of 5 min was optimized for the CPC variants described here and in the associated primary research. For other proteins, the timing may need to be optimized accordingly.
  g. Acquire an image (t = 5 min) using the transmission light channel of your microscope. If the protein of interest is fluorescently labeled also acquire the corresponding wavelength.
  h. Assess droplet formation as in step e.
  i. Repeat steps a-h over a range of various protein and salt concentrations to record phase diagrams.
2. Examine the effect of macromolecular crowding on protein *in vitro* LLPS.
  a. Calculate the volumes required to achieve the desired protein and salt concentrations.
  b. Prepare 2 separate Eppis for each reaction.
    i. Eppi 1: Add the volume of your protein as well as the appropriate volume of SEC buffer.
    ii. Eppi 2: Add the appropriate volumes of “NO salt buffer” and additives, mix by pipetting up and down several times.
  c. Transfer solution from Eppi 2 into Eppi 1 and resuspend by pipetting up and down 15x.
  d. Immediately add the solution into a well of an 8-well μ-chamber slide and start the timer.
  e. Incubate 5 min.
    ***Note:*** The incubation time of 5 min was optimized for the CPC variants described here and in the associated primary research. For other proteins, the timing may need to be optimized accordingly.
  f. Acquire an image using the transmission light channel of your microscope. If the protein of interest is fluorescently labeled also acquire the corresponding wavelength.
  g. Assess droplet formation as in 1e.

### Electroporation of recombinant proteins into living cells

**Timing: 3 days**

**Timing: 1–3 h depending on experimenter’s experience and sample number (for step 3)**

**Timing: 6 h (for step 4)**

**Timing: 2 days (for step 5)**

This technique allows the delivery of purified, recombinant proteins into mitotic HeLa cells and subsequent analysis of their localization by immunofluorescence. Total delivery levels are assessed by western blotting.

**CRITICAL:** Make sure the cells are readily prepared as described in the section cell preparation for electroporation.

***Note:*** Electroporation may also be performed on asynchronous or differently synchronized cells.

3. Electroporation.
  **CRITICAL:** Spin protein after thawing for 15 min at 4°C and 20,000 × *g* in a table top centrifuge to remove potential aggregates.
  ***Note:*** The following buffers/ solutions should be placed in a 37°C bead or water bath for pre-heating: PBS, complete DMEM, buffer E, buffer R, Trypsin/EDTA.
  a. Set up NEON electroporation devices (Figures 4A and 4B).
    ***Alternative:*** There are different commercially available electroporation devices available, e.g., the X- and Y-electroporation units by Lonza for electroporation of suspension or adherent cells, respectively.

**Figure 4:**
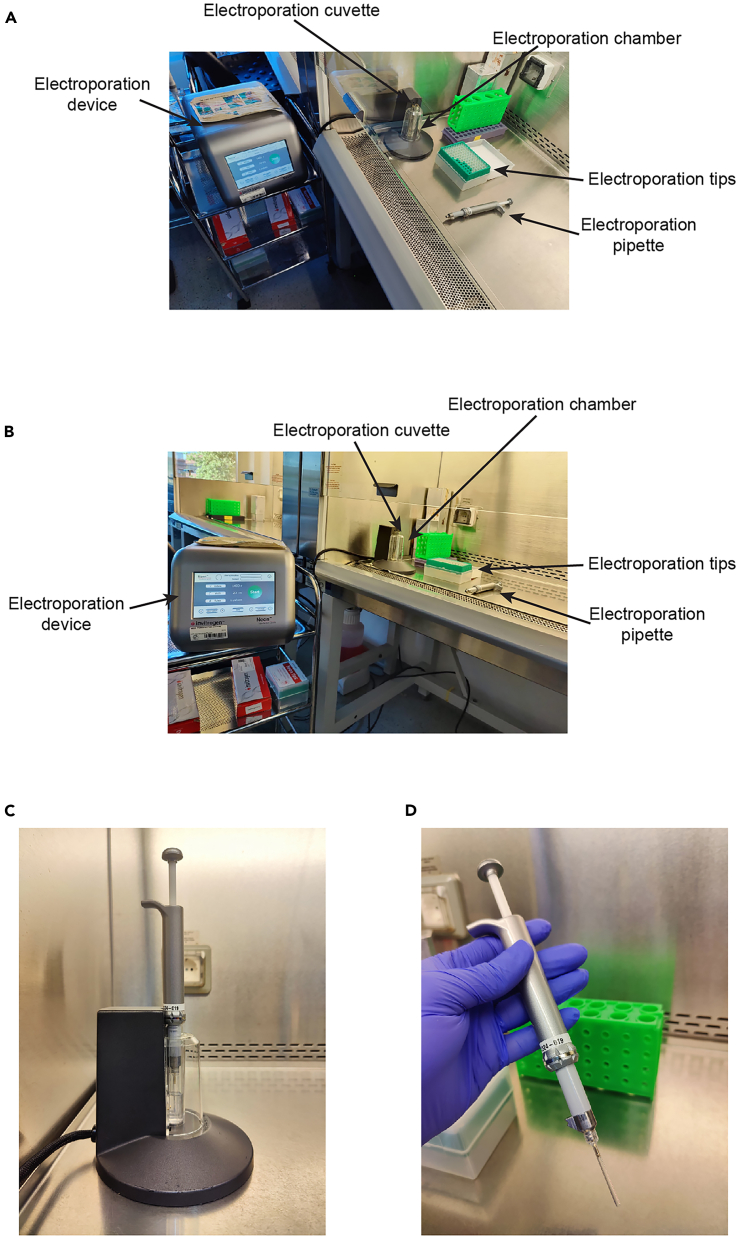
Electroporation setup (A) Top view of the electroporation setup. (B) Side view of the electroporation setup. (C) Side view of the assembled electroporation chamber. The chamber is filled with buffer E and the electroporation tip is inserted. (D) Assembled electroporation pipette.
  b. Harvest cells by mitotic shake-off and transfer all cells to a 50 mL Falcon tube.
    ***Note:*** After transfer, the cell culture dish can be additionally rinsed with PBS to ensure the collection of all mitotic cells.
  c. Pellet cells by centrifugation at 500 × *g* for 5 min.
  d. Carefully remove the supernatant and wash cells with 50 mL PBS pre-warmed to 37°C.
  e. Pellet cells by centrifugation at 500 × *g* for 5 min.
  f. Carefully remove the supernatant, resuspend cells in 1 mL PBS pre-warmed to 37°C and count cells (here we use the TC20 automated Cell Counter).
    i. Mix 10 μL of cell suspension with 10 μL of Trypan Blue.
    ii. Transfer 10 μL to a counting slide.
    iii. Insert slide into cell counter and count cells.
  g. Calculate the volume required for 1–3 × 10^6^ cells and transfer it to a fresh 1.5 mL Eppendorf tube.
  h. Centrifuge for 5 min at 500 × *g* and prepare for electroporation.
  i. While centrifuging: For every electroporation, fill up a 15 mL Falcon tube with PBS pre-warmed to 37°C.
    ***Note:*** Place the Falcon tubes in an incubator or water bath to keep the PBS at 37°C.
  j. Insert Electroporation cuvette into Electroporation chamber and add 3 mL of Buffer E (Figure 4C).
    ***Note:*** One Electroporation cuvette can be used for up to 10 electroporations.
  k. Carefully remove the supernatant and resuspend cells in 80 μL buffer R.
    **CRITICAL:** Do not leave the cells in buffer R for an extended period of time.
  l. Dilute proteins in buffer R to a concentration of 30 μM in a total volume of 40 μL.
  m. Add 40 μL of protein diluted in buffer R to 80 μL of cells in buffer R, for negative control just add 40 μL buffer R.
  n. Carefully take up cell/ protein slurry with the electroporation tip (Figure 4D).
  o. Insert the electroporation tip in the electroporation chamber (Figure 4C).
    **CRITICAL:** Avoid air bubbles in the tip!
  p. Apply one electric pulse of 1400 V for 20 ms (problem 2).
  q. Immediately transfer electroporated cells to 15 mL Falcon tube filled with PBS pre-warmed to 37°C.
  r. Repeat steps k-q for the remaining samples.
    ***Note:*** One electroporation tip can be used for two consecutive electroporations.
  s. Remove excess of protein and wash cells (problem 3).
    i. Pellet cells by centrifugation for 5 min at 500 × *g*.
    ii. Carefully remove supernatant and resuspend cells in 5 mL 0.05% Trypsin/EDTA pre-warmed to 37°C.
      ***Note:*** After centrifugation the cell pellet may be invisible. In this case, maintain some PBS to avoid loss of cells.
    iii. Incubate for 5–7 min at 37°C, e.g., in a cell culture incubator.
    iv. Fill the Falcon tube up to 15 mL with pre-warmed PBS and spin for 5 min at 500 × *g*.
    v. Carefully remove the supernatant and resuspend cells in 15 mL PBS pre-warmed to 37°C.
    vi. Centrifuge for 5 min at 500 × *g* and carefully remove the supernatant.
  t. Resuspend cells in complete DMEM without antibiotics and seed them in a multi-well plate containing at least one coverslip.
    ***Note:*** The number of coverslips added into the multi-well plate depends on the number of stainings planned in the subsequent immunofluorescence (4. Immunofluorescence).
  u. Recover cells for at least 30 min in presence of Nocodazole (3.3 μM) and MG132 (10 μM) to maintain mitotic cells before proceeding with immunofluorescence and western blot analysis (problem 4).
    ***Note:*** Centrifugation for 5 min at 500 × *g* facilitates attachment of cells.
    ***Note:*** We have allowed electroporated cells to recover for a maximum of 4 h. Longer recovery times are possible but we recommend to assess how extended recovery time influences cellular protein levels and cell viability.
  v. Process the cells for immunofluorescence (see section 4 Immunofluorescence) and western blotting (see section 5 Mitotic cell lysate preparation and western blotting) immediately after the desired recovery time.
4. Immunofluorescence.
  ***Note:*** All steps described are carried out at 20°C–25°C.
  a. Pre-extraction and fixation.
    i. Pre-extract cells with 0.5% Triton-X in 1x PHEM + microcystin (100 nM) for 5 min.
    ii. Fix in 4% PFA in 1x PHEM for 20 min.
  b. Wash coverslips.
    i. Rinse 2x quickly with 0.1% Triton-X in 1x PHEM (PHEM-T).
    ii. Rinse 1x quickly in PHEM.
      **Pause point:** Coverslips can be stored in PHEM at 4°C for up to 7 days prior to antibody staining.
  c. Block coverslips with 5% Boiled Goat Serum (BGS) for 1 h.
  d. Prepare primary antibody solution.
    i. Dilute antibodies (see key resources table for antibody dilutions) in 2.5% BGS-PHEM-T.
    ii. Add 30 μL of antibody solution per coverslips and incubate 2 h.
  e. Wash coverslips 3x for 5 min with PHEM-T.
  f. Prepare secondary antibody solution.
    i. Dilute antibodies 1/200 in 2.5% BGS-PHEM-T.
    ii. Add DAPI to a final concentration of 0.5 mg/mL.
    iii. Add 30 μL of antibody solution and incubate 1 h.
  g. Wash coverslips 2x for 5 min with PHEM-T.
  h. Wash coverslips for 5 min in PHEM.
  i. Mount coverslips onto microscope slides using Mowiol.
5. Mitotic cell lysate preparation and western blotting.
  a. Harvest mitotic cells by resuspending them 10x in the complete DMEM present in the well using a P1000 pipette.
  b. Transfer cells suspension to an appropriate vial (e.g., 1.5 mL or 2 mL Eppendorf tube).
  c. Pellet cells by centrifugation at 500 × *g* for 5 min.
    ***Note:*** Check with the microscope if all cells have detached during step 5a. If necessary, the well can be rinsed with PBS.
  d. Carefully remove the supernatant and wash cell pellet by resuspending in 1 mL PBS.
  e. Repeat step c.
  f. Carefully remove the supernatant and thoroughly resuspend cells in one pellet volume lysis buffer.
  g. Lyse cells by 3 repeated freeze-thaw cycle in liquid nitrogen.
    i. Flash-freeze cell suspension in liquid nitrogen for at least 30 s.
    ii. Remove Eppendorf tube containing the frozen cell suspension and thaw it on ice. Depending on the pellet size this takes at least 5–10 min.
    iii. Repeat steps i-ii twice.
  h. Clarify the lysate by centrifugation at 20,000 × *g* for 30 min at 4°C using a table top centrifuge.
  i. Estimate the total protein concentration using the A280 1Abs = 1 mg/mL option of a NanoDrop2000 device.
    ***Alternative:*** The protein concentration can also be measure using Bradford or BCA protein assays.
  j. Add SDS-PAGE sample buffer to a final concentration of 1x.
  k. Resolve proteins using SDS-PAGE.
    i. Load 50–100 μg per condition and well.
    ii. Run gel at 200 V and a constant ampere limit of 25 mA.
  l. Electrotransfer proteins at 100 V for 100 min onto a nitrocellulose membrane.
    ***Alternative:*** Proteins may be transferred onto a polyvinylidene difluoride (PVDF) membrane after pre-activation with 100% methanol for 3 min.
  m. Verify successful transfer by Ponceau S staining.
    i. Wash membrane for 5 min in TBS-T.
    ii. Incubate with Ponceau S for 3–5 min.
    iii. Wash away the excess of Ponceau S by rinsing in ddH_2_O.
    iv. Remove all Ponceau S by washing twice for 10 min in TBS-T.
      ***Note:*** Ponceau S staining does not work with PVDF membranes.
  n. Block the membrane for 30–60 min with 5% milk in TBS-T.
  o. Dilute primary antibody in 5% milk-TBS-T and incubate for 16–20 h at 4°C.
  p. Wash the membrane 3x for 5 min in TBS-T.
  q. Dilute secondary antibody coupled to horseradish-peroxidase in 5% milk-TBS-T and incubate 60 min at 20°C–25°C.
  r. Wash the membrane 3x for 5 min in TBS-T.
  s. Use the ECL Prime Western Blotting Detection Reagent to visualize bands by chemiluminescence.

## Expected outcomes

Purification of the different recombinant CPC variants should result in a stoichiometric trimeric complex after size-exclusion chromatography. Ideally, the DNA:protein ratio of the final purified product ranges around 0.5–0.8. Typical yields are 3–5 mg of protein per 1 L of expression culture (Figure 2).

The outcome of an LLPS experiment *in vitro* strictly depends on the chosen solvent conditions. Usually, these experiments are carried out at protein and salt concentrations considered physiological. However, we strongly recommend testing LLPS propensity *in vitro* not only in aqueous buffer but also in presence of diluted cytomimetic media. For example, the addition of whole cell bacterial or mammalian lysates prevented phase-separation of the CPC *in vitro*.^1^ The same effect may be observed upon addition of yeast lysates^30^ and different additives, e.g., FBS or lysozyme. Instead, inert crowding agents such as PEG3350 or Ficoll400 are expected to enhance *in vitro* LLPS most likely through volume exclusion effects.

The goal of our study was to assess whether LLPS or binding interactions were predictive of localization *in vivo*. As the study demonstrates that binding is the dominant determinant of localization, we expect the wild-type version of the electroporated recombinant proteins to correctly localize to centromeres and binding-deficient mutants not to localize. Our original study shows LLPS is not a predictive driver of centromere localization. For this assessment we expect the cellular levels of electroporated proteins to be comparable among conditions and ideally to match the levels of their endogenous counterparts.

## Quantification and statistical analysis

To quantify fluorescence intensities from immunofluorescence experiments, 16-bit TIFF images were exported from the Slidebook software and cropped to one cell per image. The analysis was performed using a customized MACRO in ImageJ/Fiji.

Otsu’s method was applied to threshold the reference channel (CENP-C or Aurora B) and the DAPI channel to create regions of interest (ROIs) of the centromeres and mask segmentation, respectively. Binary “Dilate” and “Fill Holes” operations were applied to the mask to fit the ROIs inside the mask area. Mean fluorescence intensities for each protein and the reference were calculated from the ROIs segmentation in their respective channels. Mean background intensities were calculated from all pixels which are part of the mask but not the ROIs, and subtracted from the protein mean fluorescent intensities. Intensity measurements were saved as .csv files and imported into Excel. Here, background-corrected mean fluorescence intensities were multiplied by the area of the ROIs to obtain the centromeric total fluorescence signal per cell for each protein and the reference. These values were then plotted in GraphPad Prism as whole cell total fluorescent intensities or normalized whole cell fluorescent intensities after calculating the ratio of protein of interest/ reference and normalization to the median of the control condition.

Statistical analysis was performed with a rank sum, nonparametric test comparing two unpaired groups (Mann-Whitney test) in GraphPad Prism.

## Limitations

We acknowledge that the use of whole cell lysates derived from bacteria and mammalian cells as cytomimetic media may have unexpected side effects. We cannot completely rule out the possibility that the target protein may get modified in unpredictable ways that may also change its phase properties.

Secondly, for studying the role of LLPS for CPC recruitment to centromeres, endogenous Aurora B activity is pivotal and we had to carry out experiments without depletion of the endogenous CPC. This may result in subunit exchange of endogenous CPC and the exogenous CPC targeting module potentially masking interpretation of data. However, we demonstrated in our original paper that electroporation of the exogenous CPC targeting complex does not negatively affect the localization of endogenous CPC. Additionally, we did not find evidence of extensive subunit exchange *in vitro*.

We cannot exclude that some proteins may prove unsuitable for electroporation as they may only get delivered in form of intracellular aggregates or not localize correctly to their designated subcellular compartment.

## Troubleshooting

### Problem 1

Cell preparation for electroporation (Step 7f).

Inefficient G2-phase release and mitotic cell enrichment. After a 16–20 h RO3306 treatment, cells should look relatively flat and bigger in size compared to asynchronously growing cells (Figures 3A and 3C). Ideally, the mitotic index of the entire dish is close to 0%. If cells look rather asynchronous the RO3306 may not be working properly (Figure 3B).

### Potential solution

RO3306 should be stored long-term at - 80°C. The drug seems to be sensitive to multiple freezing and thawing cycles. After aliquoting the stock solution, immediately flash-freeze in liquid nitrogen and transfer to - 80°C. Do not re-use already used aliquots.

### Problem 2

Electroporation of recombinant proteins into living cells (Step 3p).

Poor electroporation efficiency. The recombinant protein gets only poorly or not at all delivered into cells.

### Potential solution

Optimize the electroporation parameters by varying the voltage amplitude, pulse width or number of the pulse(s).

### Problem 3

Electroporation of recombinant proteins into living cells (step 3s).

Protein aggregates and sticks to the cell membrane upon electroporation.

### Potential solution

• Increase the incubation time in 0.05% Trypsin/EDTA for up to 10 min.
• Try 2 consecutive Trypsin/EDTA wash cycles.
• Purify a new batch of protein. In some cases, the age of the protein may play a role and extended storage at - 80°C may make it unusable for electroporation.

### Problem 4

Electroporation of recombinant proteins into living cells (Step 3u).

Increased cell mortality after electroporation.

### Potential solution

• Optimize (decrease) voltage amplitude. Some cell lines are more sensitive to the high voltage applied during the electroporation than others. Parameters should be optimized so to achieve a good balance between protein delivery and cell viability.
• Do not add antibiotics to the cell culture medium during the recovery phase.

## Resource availability

### Lead contact

Further information and requests for resources and reagents should be directed to and will be fulfilled by the lead contact, Andrea Musacchio (andrea.musacchio@mpi-dortmund.mpg.de).

### Technical contact

Questions about the technical specifics of performing the protocol should be directed to and will be answered by the technical contact, Marius Hedtfeld (marius.hedtfeld@mpi-dortmund.mpg.de).

### Materials availability

This protocol did not generate new reagents or materials. All resources described in the associated primary research are available from the lead contact.

### Data and code availability

This protocol did not generate new datasets. Essential primary data associated to the associated primary research are available from the lead contact or the Mendeley repository (https://doi.org/10.17632/srwv7hcwd5.1).

## Acknowledgments

M.H. and A.M. acknowledge funding from the Deutsche Forschung Gemeinschaft (DFG)’s Collaborative Research Centre 1093 “Supramolecular Chemistry on Proteins.” A.M. also acknowledges funding from the Max Planck Society, the European Research Council (ERC) Synergy Grant 951430 (BIOMECANET), the Marie-Curie Training Network DivIDE (project number 675737), the DGF’s Collaborative Research Centre 1430 “Molecular Mechanisms of Cell State Transitions,” and the CANTAR network under the Netzwerke-NRW program. The graphical abstract was created with BioRender.

## Author contributions

Conceptualization, M.H. and A.M.; investigation, M.H.; funding acquisition, A.M.; project administration, A.M.; supervision, A.M.; validation, M.H. and A.M.; visualization, M.H. and A.M.; writing – original draft, M.H. and A.M.; writing – review and editing, M.H. and A.M.

## Declaration of interests

The authors declare no competing interests.
